# Symmetrical Bicuspidization Minimizing Use of Pericardium for Unicuspid Aortic Valve With Aortic Regurgitation

**DOI:** 10.1016/j.atssr.2026.04.027

**Published:** 2026-05-05

**Authors:** Yutaka Okita, Ryo Kawabata

**Affiliations:** Cardio-Aortic Center, Takatsuki General Hospital, Kosobe, 1-3-13, Takatsuki, Osaka, Japan 569-1192

Reply to the Editor

We appreciate Dr Jasinski’s comments about our article.^1^^,^^2^ We concur with the concept that the unicuspid aortic valve is a variant of a bicuspid valve; however, we no longer apply the Sievers classification because the unicuspid aortic valve is one of the lesions within a broad spectrum of congenital anomalies of the aortic valve. Symmetrical bicuspidization has several advantages over asymmetrical repair^3^—better hemodynamics and technical simplicity. The essential part of this technique is making the neocommissure, which should be at the 180° angle opposite to the normal commissure and at the same height as the normal one. Use of the autologous pericardium raises some concerns about late deterioration. We tried to preserve the free margin tissue, which is more suitable than pericardium for valve opening and closing, with some shaving. The valve leaflet required augmentation of tissue volume to raise the height of the neocommissure, and we minimized use of the pericardium at the belly of the leaflet. We regret that the rigid internal remodeling annuloplasty ring cannot achieve a similar symmetrical cusp and identical commissure shape of bicuspidization.
